# Subthalamic beta peak power ratio as an electrophysiological marker for deep brain stimulation contact selection in Parkinson’s disease

**DOI:** 10.1186/s42466-025-00441-9

**Published:** 2025-10-28

**Authors:** Victoria D. M. Molinari, Matthias Sure, Rachel K. Spooner, Bahne H. Bahners, Alfons Schnitzler, Esther Florin, Christian J. Hartmann

**Affiliations:** 1 Institute of Clinical Neuroscience and Medical Psychology, Medical Faculty and University Hospital Düsseldorf, Heinrich Heine University Düsseldorf, Düsseldorf, Germany; 2 Department of Neurology, Center for Movement Disorders and Neuromodulation, Medical Faculty, Heinrich Heine University Düsseldorf, Düsseldorf, Germany

**Keywords:** Parkinson’s disease, Deep brain stimulation, Local field potentials, Beta activity

## Abstract

**Background:**

Previous studies have demonstrated that patients with Parkinson’s disease (PD) exhibit pathologically increased beta band activity (12–35 Hz) in the basal ganglia, which peaks at an individual frequency and correlates with symptom severity. The purpose of this study was to determine whether different beta peak measures can serve as predictors for deep brain stimulation (DBS) contact selection.

**Methods:**

Subthalamic local field potentials were acquired from 27 patients with PD (8 female, 59.0 ± 8.9 years) with (ON) and without (OFF) dopaminergic medication. Peak amplitudes and frequencies were detected in the low (12–20 Hz) and high beta band (21–35 Hz), and their predictive value for the motor symptom improvement, the therapeutic window and the optimal stimulation contact were analyzed.

**Results:**

In particular, the power ratio of the highest low beta peak ON versus OFF medication explained 23.7% of the variance in the therapeutic window.

**Conclusion:**

Our results demonstrate that beta peak measures can serve as valuable markers to estimate contact selection to achieve an optimal DBS outcome in patients with PD.

**Trial registration:**

Not applicable.

**Supplementary Information:**

The online version contains supplementary material available at 10.1186/s42466-025-00441-9.

## Background

Deep brain stimulation (DBS) is an effective treatment for patients with Parkinson’s disease (PD) [1]. The clinical outcome of DBS depends on the choice of DBS contact and stimulation parameters [2]. As the contact levels of DBS electrodes can be divided into segments, the potential for more targeted stimulation has increased [3]. Despite the advantages of this approach, including a reduction in adverse effects and an extended therapeutic window, the process of stimulation adjustment in monopolar testing has become more complex and time-consuming [3–5]. Thus, the identification of physiological markers that facilitate DBS adjustment is necessary to advance DBS programming.

DBS electrodes can also be utilized to record local field potentials (LFP) from target areas [6]. Increased beta band activity (12–35 Hz) in the subthalamic nucleus (STN) correlates with the severity of rigidity and bradykinesia which are clinical hallmarks of PD [7]. A reduction in beta power leads to improved mobility and can be achieved through treatment with levodopa or DBS [8–10]. The amount of stimulation-induced beta power suppression at a particular DBS contact was associated with motor symptom improvements [11]. In addition, stimulation of the contact with the highest beta activity provided the best clinical outcome in the majority of patients [4, 12]. Furthermore, this contact frequently exhibited the largest therapeutic window (TW, minimal stimulation amplitude eliciting clinical benefits vs. side effects) [4].

Taken together, the identification of physiological markers that facilitate DBS adjustment is of fundamental importance. Considering the aforementioned findings on beta activity and the fact that the increase in beta activity is often not broadly distributed, but usually forms an individual peak at a specific frequency [13], we hypothesized that different beta peak measures could serve as predictors for DBS contacts with the broadest TW and the highest clinical efficacy. The present study therefore focused on the amplitude, frequency, standard deviation and power of these beta peaks to evaluate their potential as predictors of TW and the choice of stimulation contact with the best clinical outcome in subthalamic DBS.

## Methods

### Subjects, surgery and experimental setup

This study included 27 patients (8 female, median age 59.0 ± 8.9 years) with PD who were selected for STN DBS surgery based on the guidelines of the German Society of Neurology [14]. DBS electrodes were bilaterally implanted at the University Hospital Düsseldorf, targeting the dorsal STN (see additional file 1). The surgery was performed in two stages to enable LFP recordings from externalized DBS leads. First, DBS electrodes were implanted and externalized via the Abbott extension 6373 (Abbott Laboratories, Lake Bluff, United States). LFPs were measured 1–3 days postoperatively while the DBS electrode extensions were connected with an electroencephalogram amplifier integrated into a 306-channel magnetoencephalography system (Elekta Neuromag, Finland) [15, 16]. Only the LFP recordings were analyzed in this study.

Resting activity (eyes open) was measured with (ON) and without (OFF) dopaminergic medication in three consecutive blocks of 10 min each. One patient could be measured only in the ON state and one only in the OFF state. During the session, motor impairment was assessed using the MDS-Unified Parkinson’s Disease Rating Scale Part III (MDS-UPDRS-III) [17, 18]. One day after the recording, the pulse generator was implanted during a second surgical procedure. Since we used the raw data from a previous study, please refer to Sure et al. (2021) for details on data acquisition [15].

6–24 months after implantation, the TW of (mean ± standard deviation: 11 ± 5) DBS contacts was determined in 20 patients in a monopolar review [19]. For 4 of these patients, monopolar review data were only available for one electrode. The TW was defined as the range between the lowest stimulation amplitude that caused a relevant clinical improvement and the lowest stimulation amplitude that caused immediate side effects [3]. We defined the contact used for chronic stimulation 6–24 months after implantation as the best clinical contact (BCC) per electrode. One electrode was deactivated at the time of the examination, resulting in 53 BCCs in 27 patients.

### Signal processing and detection of beta peaks

Data were processed using MATLAB (version R2018b; MathWorks, Natick, United States) and the Brainstorm toolbox (version 12-Aug-2022) [20]. Each electrode has eight contacts in four planes, resulting in eight LFP channels, which were re-referenced to the mean of all LFP channels per patient. At the two central levels, the ring contact is divided into three segments. To address the possibility of omnidirectional stimulation at the 2nd and 3rd contact level, we developed a method to create two artificial LFP channels per electrode. The signals from the three directional channels were averaged for each height, increasing the number of LFP channels per electrode from eight to ten. 20 LFP channels were examined per patient, considering both hemispheres. As in the case of Sure et al. (2021), all LFP data were reviewed and cleaned for artifacts [15]. Out of 540 LFP channels (20 per patient), 42 bad channels (17 in the OFF state and 25 in the ON state) were identified due to flat, noisy activity or multiple artifacts and were excluded from subsequent analyses. All data were subsequently down-sampled to 1000 Hz. A notch filter with a 3-dB bandwidth of 1 Hz was employed to eliminate line noise, whereas a 1 Hz high-pass filter was utilized to remove motion-related artifacts. Power spectra were generated from the LFP data and calculated using Welch’s method with a window length of 4000 msec and a window overlap ratio of 50% using the Brainstorm toolbox (version 12-Aug-2022) [20]. The Brainstorm implementation “fitting oscillations & one over f” was utilized to identify up to three peaks (henceforth referred to as “beta peaks”) between 10 and 40 Hz in the power spectra [21]. This allowed analyzing the occurrence of peaks in the low (LBB; 12–20 Hz) and high (HBB; 21–35 Hz) beta band. Previous literature was utilized to define the frequency ranges of LBB and HBB [18, 22]. Furthermore, we selected a Gaussian peak model for peak detection, with a peak width limit between 0.5 and 12.0 Hz, a minimum peak height of 3 dB (in log[Power]), and a proximity threshold of two standard deviations. The beta peak frequency, amplitude, standard deviation and power were calculated for the highest peak amplitude in the entire beta band and for the LBB only.

### Statistics

Statistical analyses were conducted using SPSS Statistics (version 29.0.0.0, IBM, Ehningen, Germany). A significance level of 5% (alpha = 0.05) was applied, and for multiple comparisons, the *p*-values were corrected using the Bonferroni method. The corrected *p*-values are provided in the text (see additional file 2 for uncorrected *p*-values).

We analyzed the number of beta peaks in the ON and OFF state and their occurrence in the LBB and HBB. For this purpose, the beta peak with the highest amplitude per LFP channel or per BCC was considered. A Wilcoxon signed rank test was used to examine the difference in the power of the highest amplitude beta peak in the OFF state before and after the administration of dopaminergic medication. Furthermore, two additional Wilcoxon signed rank tests were calculated. First, we tested whether the frequency of the highest peak differed between the ON and OFF state. Second, we compared the average power between the ON and OFF states across the entire beta band and across the LBB and HBB.

Subsequently, the amplitude, frequency and standard deviation of the highest beta peak per LFP channel were considered as predictors for the BCC and the TW. We chose the amplitude based on the correlation between beta activity and symptom severity [7]. Due to the different characteristics of LBB and HBB described in literature [6, 23, 24], we further included the frequency of the beta peak as a possible predictor. The standard deviation, which describes the spectral width of the detected peak allowed us to examine not only the impact of the peak amplitude but also the frequency selectivity of the oscillatory activity. A logistic regression was calculated using the amplitude, the standard deviation and the frequency of the highest beta peak in the ON and OFF state as predictors of BCC. We further assessed the predictive power of these variables for the TW using linear regression.

Since beta suppression has been associated with bradykinesia improvement [10], we chose to calculate the power ratio of the highest beta peak amplitude between the medication ON and OFF state (beta peak power ON/beta peak power OFF) to assess its predictive value for BCC and TW. This peak power ratio (PPR) was calculated twice. First with the power of the highest amplitude when considering the entire beta band (PPR_LBB+HBB_) and second when considering only the LBB (PPR_LBB_). PPR_LBB+HBB_ and PPR_LBB_ were used as independent variables to delineate their predictive value for the BCC and the TW. Binary logistic regressions were calculated for the prediction of the BCC, and linear regressions were used to predict the TW.

The medication-related improvement in MDS-UPDRS-III was calculated as a ratio to the OFF-state to assess whether beta peaks can reflect medication-related improvement.

To identify potential predictors of motor improvement, Pearson correlations between beta peak measures (amplitude, frequency and standard deviation in the ON and OFF state, PPR_LBB+HBB_ and PPR_LBB_) and MDS-UPDRS-III improvement were computed. According to these results, we used PPR_LBB+HBB_ and PPR_LBB_ and calculated a linear regression model with each ratio to predict MDS-UPDRS-III improvement.

## Results

### Patient details

Information on the 27 patients is shown in Table 1.

**Table 1:** Clinical features of the investigated patients

Patient	Sex	Age [years]	MDS-UPDRS-III			
			Left		Right	
			OFF	ON*	OFF	ON*
1	m	68	19	14	12	11
2	m	56	8	8	10	8
3	m	64	13	12	10	9
4	f	62	6	8	10	11
5	m	69	9	8	10	10
6	m	45	3	2	15	7
7	m	55	6	5	6	6
8	m	77	15	NA	15	NA
9	m	54	NA	5	NA	3
10	f	60	10	8	13	12
11	m	46	5	4	5	4
12	m	55	17	8	5	4
13	m	59	7	2	8	3
14	m	66	6	2	7	3
15	m	54	10	8	14	10
16	m	41	12	4	9	4
17	f	58	9	5	6	4
18	f	65	7	3	7	6
19	f	62	15	12	11	8
20	f	44	11	6	18	5
21	m	68	12	5	12	6
22	m	53	8	2	2	0
23	m	58	12	9	14	10
24	m	69	7	5	9	6
25	f	62	2	2	15	11
26	f	75	10	8	19	11
27	m	58	2	2	6	6

Notes: m = male, f = female, NA = not available, *Medication ON

### Presence and localization of beta peaks

**Fig. 1:**
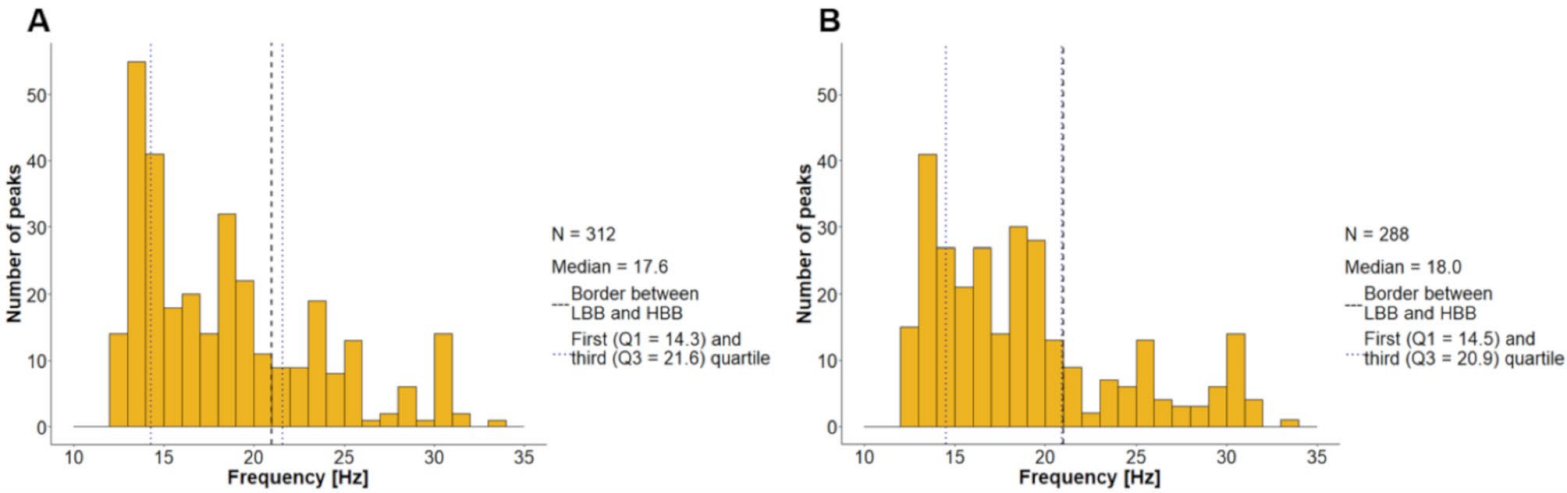
Beta peak frequency distribution. Considering the highest amplitude per LFP channel without (**A**) or with (**B**) medication

For each of the 27 patients, we determined 20 LFP channels, resulting in 540 LFP channels, of which 498 could be analyzed. To assess how reliable beta peaks can be detected in LFP recordings, we analyzed the number of beta peaks in the ON and OFF state. OFF medication, beta peaks were detected in 312 recordings (25 out of 27 patients). Among those, the beta peak with the highest amplitude was located in the LBB in 227 cases and the HBB in 85 cases (Fig. 1A). ON medication, beta peaks were observed in 288 channels (24 out of 27 patients). These peaks were found to be in the LBB in 216 cases, the remaining 72 peaks were assigned to the HBB (Fig. 1B).

Since the BCC was determined for both the left and right hemispheres, a total of 53 BCCs were identified (two for each of the 27 patients, one electrode was excluded). Beta peaks were detected in the LFP recordings of 32 BCCs in the OFF state. In 24 cases, the highest amplitude was in the LBB. ON medication, beta peaks occurred for 29 BCCs. The beta peaks with the highest amplitude were mostly in the LBB (17/29).

### Changes in beta power

**Fig. 2:**
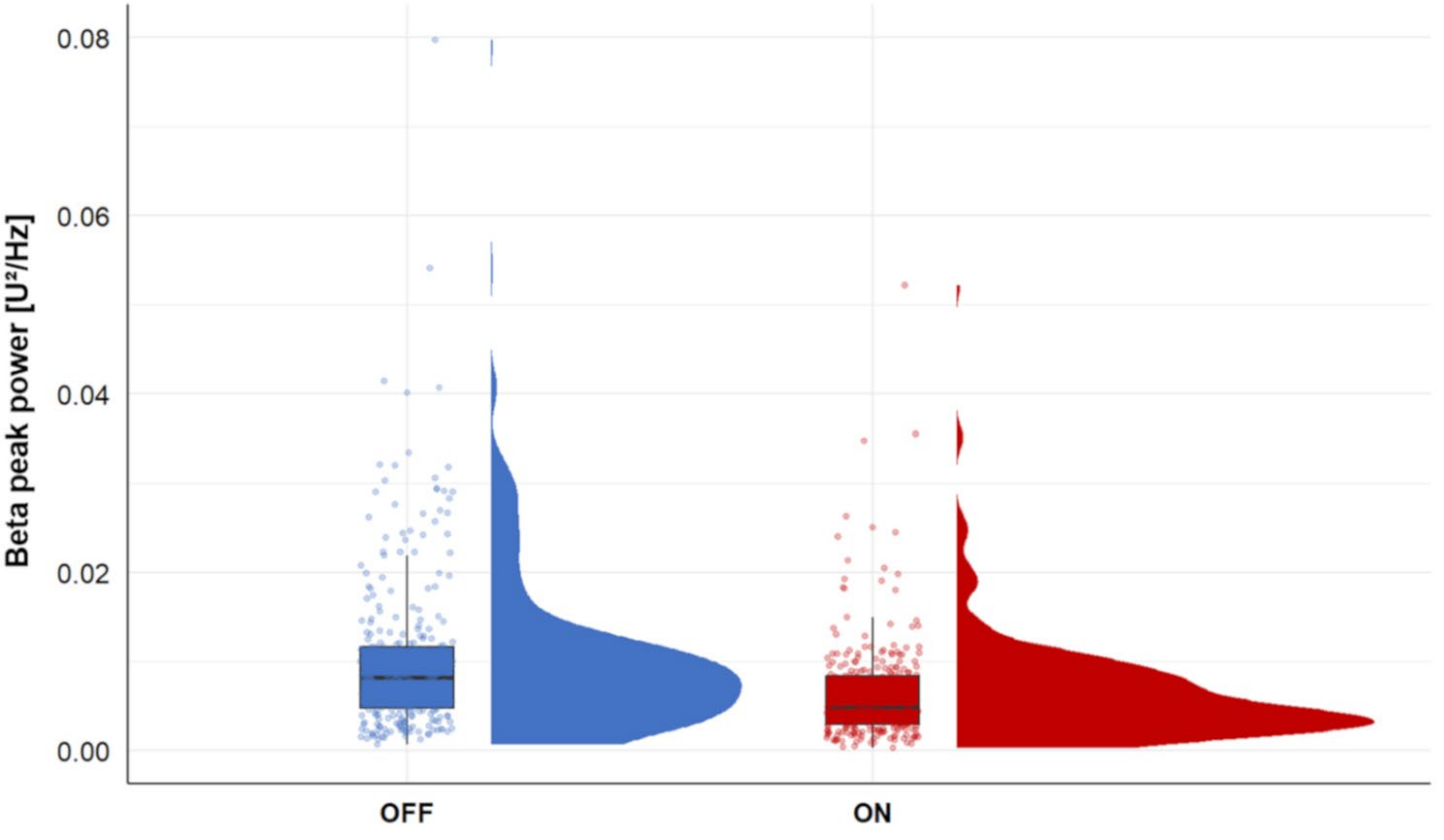
Peak power comparison. Peak power at the frequency of the highest beta peak in the OFF state per LFP channel before (OFF) and after the administration of dopaminergic medication (ON)

The power obtained in the frequency range of the highest beta peak amplitude in the OFF state significantly decreased after the administration of dopaminergic medication (Wilcoxon signed-rank test: Z = -11.068, *p* < .001) (Fig. 2). To investigate whether this result was due to a reduction in peak amplitude and not a frequency shift in the peak, we additionally considered the frequency of the highest beta peak amplitude in the ON and OFF state and the mean power, defined as the mean value of the power spectrum within the specified frequency range. The frequency of the highest beta peaks did not differ significantly between the ON and OFF states (Wilcoxon signed-rank test: Z = − 0.704, *p* = .482). A significant decrease in mean power was observed across the entire beta band (Z = -12.833, *p* < .001), LBB (Z = -10.647, *p* < .001), and HBB (Z = -12.969, *p* < .001), ON vs. OFF medication.

### Potential predictors of the BCC and TW

The following variables were examined to determine whether certain beta peak measures might facilitate the selection of DBS contact for optimal clinical outcome.

The amplitude, frequency, and standard deviation of the highest beta peak in the ON and OFF state had no predictive value for the BCC (R² = 0.066, *p* > .999), nor for the size of the TW (R² = 0.107, F (6,78) = 1.557, *p* = .513).

**Fig. 3:**
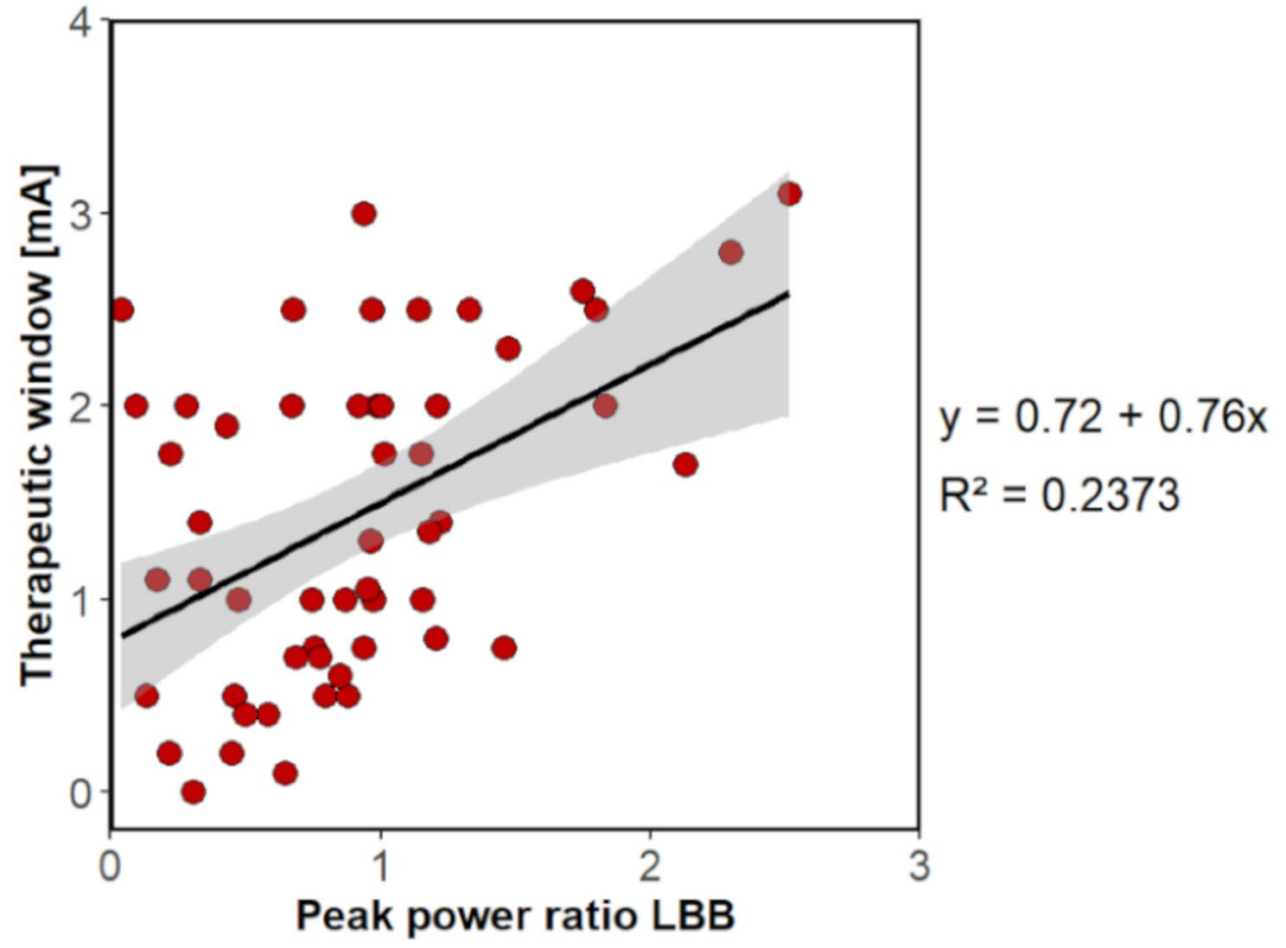
Linear regression model for predicting the therapeutic window

In turn, the PPR_LBB+HBB_ was a significant predictor of the BCC (R² = 0.064, *p* = .03) but had no predictive value for the TW (R² = 0.066, F (1,83) = 5.853, *p* = .054). Conversely, the PPR_LBB_ had no predictive value for the BCC (R² = 0.075, *p* = .063), whereas significant results were observed for the TW (R² = 0.237, F (1,52) = 16.180, *p* < .001) (Fig. 3). A post-hoc analysis revealed a positive correlation between PPR_LBB_ and TW (*r* = .487, *p* < .001, Pearson correlation). Consequently, an increase in the PPR_LBB_ is associated with an increase in the TW.

### Prediction of medication-related motor improvement

In addition to the relevance of beta peaks for DBS contact selection, we determined their predictive power for medication-related motor symptom improvement.

The frequency, amplitude, and standard deviation of the highest beta peak per LFP channel did not correlate with MDS-UPDRS-III improvement (Table 2). Significant correlations were observed between the PPRs and MDS-UPDRS-III improvement (Table 2). Consequently, the subsequent analysis focused on the prediction of the MDS-UPDRS-III improvement by PPRs.

**Table 2:** Pearson correlations between the MDS-UPDRS-III improvement and possible predictors

		PPR_LBB+HBB_	PPR_LBB_	Highest beta peak					
				Frequency		Amplitude		Standard deviation	
				OFF	ON	OFF	ON	OFF	ON
MDS-UPDRS-III improvement	r	0.347	0.303	0.139	− 0.053	− 0.021	− 0.020	0.120	− 0.007
	*p*	0.001	< 0.001	0.135	> 0.999	> 0.999	> 0.999	0.342	> 0.999
	*N*	205	153	302	271	302	271	302	271

The PPR_LBB+HBB_ (R² = 0.120, F (1,203) = 27.810, *p* < .001) and the PPR_LBB_ (R² = 0.092, F (1,151) = 15.317, *p* < .001) were statistically significant predictors of motor improvement (linear regression models).

## Discussion

Within this study, we leveraged a large LFP dataset with recordings from 540 subthalamic DBS contacts. The results confirmed that beta peaks occur in the STN of patients with PD, especially in the LBB, and that dopaminergic medication significantly reduce peak power. Our data provided further evidence that beta peaks in the STN LFP may facilitate the identification of the best clinical DBS contact. In particular, the beta peak power ratios were relevant predictors of the TW and the BCC.

### Beta peaks mainly occurred in the LBB

The majority of patients had beta peaks (25 OFF and 24 ON medication), underlining their utility as electrophysiological markers. In the ON and OFF state, more than 72% of the highest amplitude beta peaks per LFP channel were detected in the LBB. Similarly, when looking only at the BCC, we observed more peaks in the LBB than in the HBB. The predominance of peaks in the LBB and the fact that beta peaks could not be detected in every patient has already been described in previous studies [4, 25].

### Dopaminergic drugs decreased the beta peak power

Previous studies have shown that beta band power decreases with levodopa and DBS [7, 8, 26–28]. It has further been demonstrated that the beta band is more sensitive to levodopa in the low frequency range between 13 and 20 Hz than at higher beta frequencies [23, 29]. In our study the power decreased significantly at the frequency of the highest beta peak in OFF after the administration of dopaminergic medication. This power reduction could be attributed to both, a reduction in power or a shift in the peak frequency. To rule out a shift in the peak frequency, we analyzed the mean power and observed a decrease in the medication ON state across the entire beta band as well as individually for the LBB and HBB. Furthermore, our data revealed no difference in the frequency of the highest beta peak amplitude between the ON and OFF state. Therefore, a decrease in beta peak power rather than a significant frequency shift of the beta peak can be assumed. These findings confirm previous data indicating a reduction in beta peak power due to dopaminergic medication [30].

### Predictive value of beta peak power ratios for medication-related motor improvement

For patients, individual improvements in motor impairment and thus an increase in quality of life under therapy (medication and DBS) is of particular importance [1]. The results indicated that neither the frequency, amplitude, nor standard deviation of the highest beta peak per LFP channel in the ON and OFF status correlated with MDS-UPDRS-III improvement. However, the PPRs correlated with the MDS-UPDRS-III improvement and were statistically significant predictors. The PPR_LBB+HBB_ explained 12% and the PPR_LBB_ explained 9.8% of the variance in MDS-UPDRS-III improvement and thus served as markers of dopaminergic medication-related improvement in motor symptoms. A positive correlation between beta power and clinical impairment as well as a reduction in beta power due to dopaminergic medication has already been demonstrated in studies [7, 23]. Our data complement those findings by indicating that beta peak power ratios are of predictive value for the MDS-UPDRS-III improvement. Furthermore, the response to levodopa is a selection criterion for patients undergoing DBS therapy, as these patients demonstrated enhanced clinical outcomes with DBS [31, 32]. As PPRs are related to levodopa-induced MDS-UPDRS-III improvement, they might also serve to estimate the response to DBS. Indeed, in our study, the PPR_LBB_ was associated with the size of the TW.

### Determination of the BCC and the TW with beta peaks

Algorithms based on LFP data are being investigated to predict the contact with the best clinical outcome [9, 11, 12] and the DBS contact with the highest beta activity often has the largest TW [4]. Therefore, the initial analysis focused on whether contact with the highest beta peak amplitude during ON or OFF medication could serve as a predictor for the BCC and TW. Our data revealed that neither the amplitude nor the corresponding frequency or standard deviation of this beta peak were suitable predictors. In the present study, the power ratio between the highest beta peak amplitudes ON vs. OFF occurring in the LBB (PPR_LBB_) was identified as the most robust predictor for determining the TW and explained 23.7% of the variance in the TW. Tinkhauser et al. (2018) demonstrated that contacts with the largest TW corresponded to the BCC in most cases [4]. Since the size of the PPR_LBB_ correlated positively with the TW, it could be assumed that contacts with a high ratio are more likely to have high clinical effectiveness. Although the PPR_LBB_ was not a significant predictor for the BCC. Conversely, the PPR_LBB+HBB_ had no predictive value for the TW, but showed statistical significance in determining the BCC. This could be related to the fact, that HBB power is particularly reduced by DBS, while dopaminergic medication rather suppresses LBB power [23, 29].

Various factors can cause the deviation of the TW and BCC predictions. In a monopolar review, a decision must be made in favor of one contact, even if several contacts have a high TW [4]. Delayed effects cannot be considered, and other factors such as cooperation and residual medication effects, may also influence the choice of contact [19]. Thus, the monopolar review has certain weaknesses in evaluating the stimulation contact, which are reflected in the definition of the BCC and make it difficult to determine.

The observation that only the PPRs possess predictive value, while the amplitude, standard deviation, and frequency do not, indicates that the change between two states is clinically relevant, rather than the absolute values. Furthermore, ratios eliminate external factors like impedances, are robust against systematic errors and facilitate comparisons between individuals.

Our findings suggest that beta peak power ratios may be integrated into future adaptive DBS algorithms, reducing reliance on lengthy monopolar reviews. Using novel sensing-enabled neurostimulators [10], a levodopa challenge could be performed alongside LFP recordings to determine the PPRs. However, it should be noted that the correlation between PPRs and BCC is low, which limits their direct and sole clinical utility. We consider PPRs to be additional factors that, alongside other parameters, may contribute to improved and simplified selection of stimulation contacts in the future.

### Limitations

In terms of limitations, it should be noted that the patient’s attention could be impaired due to fatigue or exhaustion caused by the anesthetics used during the surgical procedure [33]. Furthermore, the data were collected between one and three days after surgery, a period during which the stun effect may have influenced beta activity and thus the results reported here [34]. Studies have demonstrated that beta activity and beta peak frequency remain constant over several months following DBS surgery [35]. Nevertheless, taking chronic measurements weeks or months after surgery to rule out the stun effect could lead to changes in predictive accuracy.

## Conclusions

Our findings indicate that the majority of patients with PD in our study had a beta peak within the STN. The occurrence of these peaks was primarily observed in the LBB, and their power decreased in response to dopaminergic medication. We show that beta peak power ratios can estimate the response to levodopa in terms of MDS-UPDRS-III improvement. In addition, the PPR_LBB+HBB_ predicted the BCC, while the PPR_LBB_ emerged as a significant predictor of the TW. With novel sensing-enabled neurostimulators, LFP readout has become accessible in clinical DBS care. In the future, PPRs could contribute to further optimizing the choice of stimulation contact, in addition to other parameters. The predictive power of beta peak measures should be further investigated in larger cohorts to advance electrophysiology-based DBS programming.

## Supplementary Information

Below is the link to the electronic supplementary material.

Supplementary Material 1

## Data Availability

The data that support the findings of this study are available from the corresponding author upon reasonable request.

## Abbreviations

BCC: Best clinical contact
DBS: Deep brain stimulation
HBB: High beta band (21–35 Hz)
LBB: Low beta band (12–20 Hz)
LFP: Local field potential
MDS-UPDRS-III: MDS-Unified Parkinson’s Disease Rating Scale Part III
OFF: Without dopaminergic medication
ON: With dopaminergic medication
PD: Parkinson’s disease
PPR: Peak power ratio
PPR_LBB_: Peak power ratio considering the low beta band
PPR_LBB+HBB_: Peak power ratio considering the entire beta band
STN: Subthalamic nucleus
TW: Therapeutic window
