# Supplementary material for: Subthalamic beta peak power ratio as an electrophysiological marker for deep brain stimulation contact selection in Parkinson’s disease
Source: Neurol Res Pract. 2025 Oct 28;7(1):81. doi: 10.1186/s42466-025-00441-9 (PMC12570425; doi:10.1186/s42466-025-00441-9)
Supplement: Supplementary file 1 — Supplementary Material 1 [file 42466_2025_441_MOESM1_ESM.docx]

**Supplementary Fig. 1** Electrode position


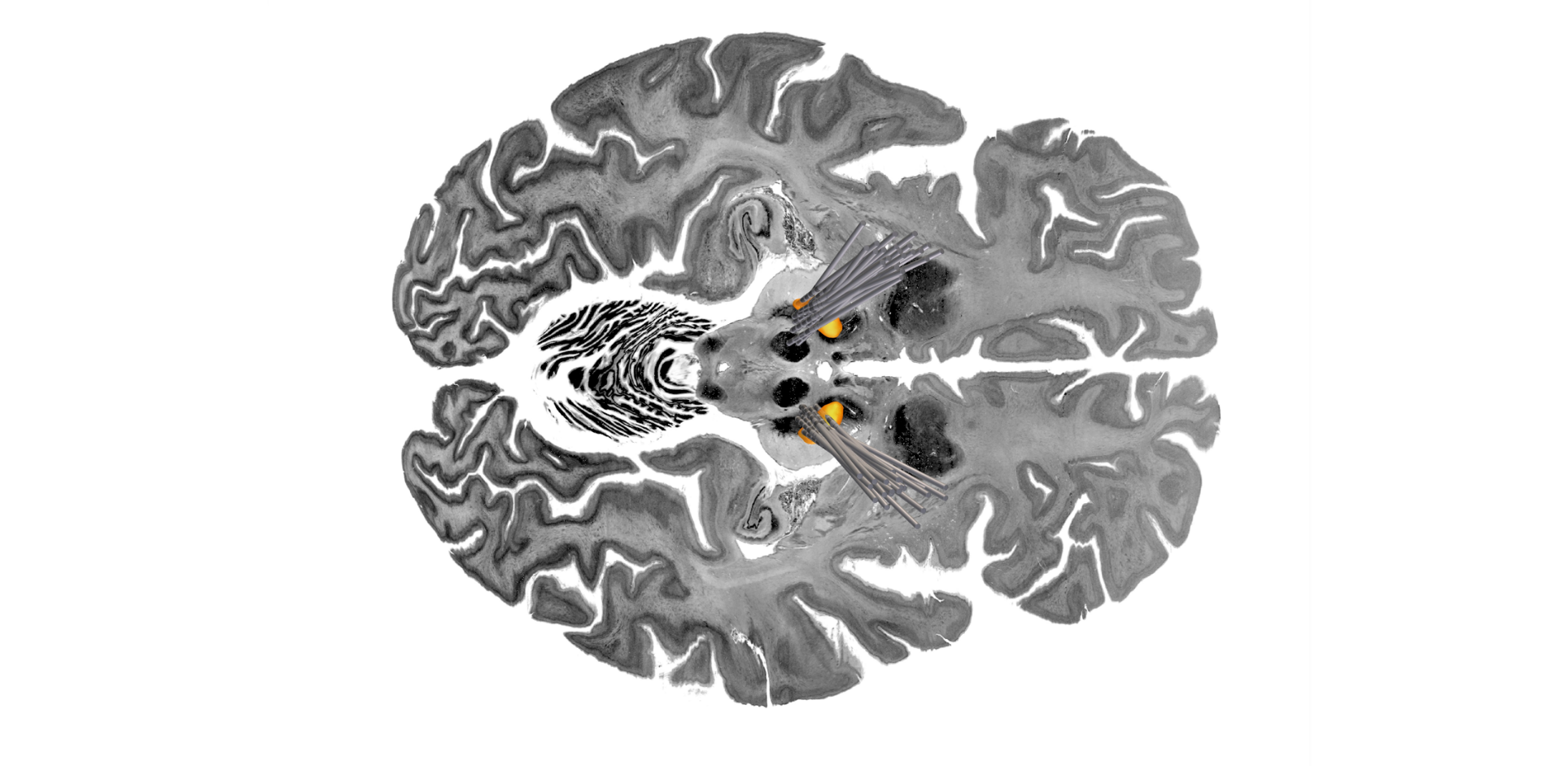


To localize the DBS electrodes with Lead-DBS v3.0 [1, 2], preoperative MRI and postoperative CT were co-registered and non-linearly warped to ICBM 2009b Nonlinear Asymmetric ('MNI') space using Advanced Normalization Tools. A brain shift correction was applied, and electrode trajectories were reconstructed using PaCER [3, 4]. For the purposes of electrode visualization, the DISTAL and Big Brain atlases were used [5, 6].

**Supplementary Table 1** Bonferroni correction of p-values in multiple comparisons

| Statistical test | Variables | Uncorrected p-value | Corrected p-value (Bonferroni) |
| --- | --- | --- | --- |
| Wilcoxon signed-rank test | - Beta power (ON medication) at the Frequency of the highest beta peak in OFF medication state - Beta power (OFF medication) at the Frequency of the highest beta peak in OFF medication state | 1.184E-9 | <.001 |
| Wilcoxon signed-rank test | - Mean beta power (entire beta band) OFF medication - Mean beta power (entire beta band) ON medication | 1.076E-37 | <.001 |
| Wilcoxon signed-rank test | - Mean beta power   (low beta band)  OFF medication   - Mean beta power   (low beta band)  ON medication | 1.8025E-26 | <.001 |
| Wilcoxon signed-rank test | - Mean beta power (high beta band)   OFF medication   - Mean beta power   (high beta band)  ON medication | 1.8336E-38 | <.001 |
| Binary logistic regression | - Dependent variable: BCC - Independent variable: amplitude, frequency, standard deviation of the highest beta peak ON and OFF medication | .342 | >.999 |
| Binary logistic regression | - Dependent variable: BCC - Independent variable: PPR_LBB+HBB_ | .01 | .03 |
| Binary logistic regression | - Dependent variable: BCC - Independent variable: PPR_LBB_ | .021 | .063 |
| Linear regression | - Dependent variable: TW - Independent variable: amplitude, frequency, standard deviation of the highest beta peak ON and OFF medication | .171 | .513 |
| Linear regression | - Dependent variable: TW - Independent variable: PPR_LBB+HBB_ | .018 | .054 |
| Linear regression | - Dependent variable: TW - Independent variable: PPR_LBB_ | .000187185014138 | <.001 |
| Pearson correlation | - MDS-UPDRS-III improvement - PPR_LBB+HBB_ | .000137118444692 | .001 |
| Pearson correlation | - MDS-UPDRS-III improvement - PPR_LBB_ | .00000034145 | <.001 |
| Pearson correlation | - MDS-UPDRS-III improvement - Highest beta peak frequency OFF medication | .015 | .135 |
| Pearson correlation | - MDS-UPDRS-III improvement - Highest beta peak frequency ON medication | .389 | >.999 |
| Pearson correlation | - MDS-UPDRS-III improvement - Highest beta peak amplitude OFF medication | .721 | >.999 |
| Pearson correlation | - MDS-UPDRS-III improvement - Highest beta peak amplitude ON medication | .747 | >.999 |
| Pearson correlation | - MDS-UPDRS-III improvement - Highest beta peak standard deviation OFF medication | .038 | .342 |
| Pearson correlation | - MDS-UPDRS-III improvement - Highest beta peak standard deviation   ON medication | .915 | >.999 |
| Linear regression | - Dependent variable: MDS-UPDRS-III improvement - Independent variable: PPR_LBB+HBB_ | 3.4145E-7 | <.001 |
| Linear regression | - Dependent variable: MDS-UPDRS-III improvement - Independent variable: PPR_LBB_ | .000137118444692 | <.001 |

**References**

1. Neudorfer, C., et al., *Lead-DBS v3.0: Mapping deep brain stimulation effects to local anatomy and global networks.* Neuroimage, 2023. **268**: p. 119862.

2. Hollunder, B., et al., *Mapping dysfunctional circuits in the frontal cortex using deep brain stimulation.* Nat Neurosci, 2024. **27**(3): p. 573-586.

3. Horn, A., et al., *Lead-DBS v2: Towards a comprehensive pipeline for deep brain stimulation imaging.* Neuroimage, 2019. **184**: p. 293-316.

4. Husch, A., et al., *PaCER - A fully automated method for electrode trajectory and contact reconstruction in deep brain stimulation.* Neuroimage Clin, 2018. **17**: p. 80-89.

5. Ewert, S., et al., *Toward defining deep brain stimulation targets in MNI space: A subcortical atlas based on multimodal MRI, histology and structural connectivity.* Neuroimage, 2018. **170**: p. 271-282.

6. Amunts, K., et al., *BigBrain: an ultrahigh-resolution 3D human brain model.* Science, 2013. **340**(6139): p. 1472-5.
